# Considerations about causality in observational studies

**DOI:** 10.1016/j.lana.2021.100135

**Published:** 2021-12-11

**Authors:** Bruna Venturin

I hereby congratulate the authors of the excellent work “Association between Ambient Temperature and Hospitalization for Renal Diseases in Brazil during 2000–2015: A Nationwide Case-Crossover Study” for their contribution in the field of health while we make some contributions and clarifications on interpretations, study design and results.^1^

Regarding the study design, the author(s) present that the data are secondary and aggregated, collected through the information system of the Brazilian Unified Health System (SUS) and call this design a "case-crossover", although it would be correct to characterize or classify the study as ecological.^2^^,^^3^ It should be noted that one of the most important criteria for talking about causality is temporality. If we managed to lower the temperature, would hospitalizations for kidney disease end? In the case of this study, the temporality of exposure to temperature was not prior to hospitalization for kidney disease, therefore, the authors make an error that we call temporal ambiguity. Bearing in mind that the effect of temperature on the renal system is not immediate, it is a problem that is triggered by continuous exposure over time.^2^^,^^3^

Regarding the calculation of attributable risk in the study and the causality conclusions exposed by the authors, such statements are incorrect. First, the authors present right in the introduction that the association and causality between temperature and kidney disease are still unknown in larger studies with higher quality designs.

Another point for discussion is about the magnitude of the relative risks (RR) found, for example: at each increase of 1 °C, the RR of the association between hospitalization for kidney disease increased by 0.09%. The question is: how important is this biologically, clinically and financially for public health programs and interventions by programs or public policies?

I would add that the study showed a greater number of hospitalizations for kidney disease among women, but it did not mention that here in Brazil, women tend to seek health services more than men.^4^ That is, the association found may be spurious or non-causal. Regarding the differences found between the regions of Brazil, it is noteworthy that the system for collecting and recording information by the Unified Health System is not equally distributed in municipalities and states and this difference found may be due to this. Registration is better in regions with better distribution of equipment such as computers and internet.^4^, ^5^, ^6^

Finally, I congratulate the authors for raising this hypothesis and for carrying out the study. I also take this opportunity to thank you for your attention.

## Declaration of interest

The author has nothing to declare.
